# Some Aspects of α-(Acyloxy)alkyl Radicals in Organic Synthesis †

**DOI:** 10.3390/molecules28227561

**Published:** 2023-11-13

**Authors:** Béatrice Quiclet-Sire, Samir Z. Zard

**Affiliations:** Laboratoire de Synthèse Organique associé au C.N.R.S., UMR 7652, Ecole Polytechnique, 91128 Palaiseau, France; beasire52@gmail.com

**Keywords:** radical additions, xanthates, α-(acyloxy)alkyl radicals, enones

## Abstract

The preparation and use of α-(acyloxy)alkyl xanthates to generate and capture α-(acyloxy)alkyl radicals is briefly reviewed. Their inter- and intramolecular additions to both activated and unactivated, electronically unbiased, alkenes, and to (hetero)aromatic rings, as well as their radical allylation and vinylation reactions are described. Application to the total synthesis of two 4-hydroxytetralone natural products is also presented.

## 1. Introduction

**Scheme 1:**
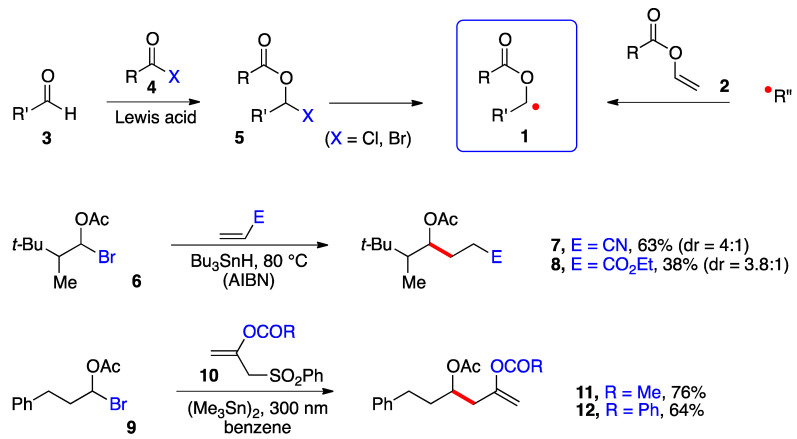
α-(Acyloxy)alkyl radicals and early examples of additions.

α-(Acyloxy)alkyl radicals of general structure **1** are key intermediates in the polymerization of vinyl esters **2**, especially vinyl acetate (**2**, R = Me); however, their use in the synthesis of small molecules has remained underdeveloped. Perhaps the main impediment to produce and capture these radicals in a synthetically meaningful setting has been the relative inaccessibility and/or lability of precursors **5** (Scheme 1). α-(Acyloxy)alkyl chlorides (**5**, X = Cl) are easier to handle and purify than the bromides (X = Br) but are poorer radical precursors [1,2]. In most cases, such derivatives are prepared and used directly. Two types of transformations are pictured in Scheme 1. In the first, a classical Giese-type addition of bromide **6** to acrylonitrile and ethyl acrylate occurs to give adducts **7** and **8**, respectively [3]. In the second, the radical from bromide **9** undergoes an addition–elimination onto alkenes **10** to give enol esters **11** and **12** [4]. Both studies relied on a stannane reagent to generate the desired radical intermediates. More recently, Glorius and co-workers generated the benzoylated ketyl radical **1** (R = Bz) from the in situ formed corresponding bromide **5** (R = Bz, X = Br) by irradiation (30 W 450 nm LED) in the presence of tris(trimethylsilyl)silane and an iridium catalyst and used a nickel catalyst to mediate its coupling with (hetero)aromatic bromides [5]. Nagib and colleagues used acetyl iodide and catalysis by zinc triflate to form in situ the geminal acetoxy iodide **5** (R = Ac, X = I) and produced the corresponding radical **1** (R = Ac) by reduction with Mn_2_(CO)_10_ in the presence of Hünig’s base (*i*-Pr_2_NEt) and irradiation with a blue LED. The radical was captured by an activated terminal alkyne or alkene in an iodine transfer Kharasch-type process [6]. In both of these more recent studies, relatively simple aldehydes **3** were used as starting materials. Only in one case was the iodide generated from a very particular ketone, namely 1,1,1-trifluoroacetone [6]. The strongly electronegative fluorine atoms protect the derived geminal acetoxy-iodide against elimination, allowing it to survive the radical addition conditions.

Another simple route to produce α-(acyloxy)alkyl radicals is by a radical addition to vinyl esters **2**. However, while numerous instances of such additions have been reported, the resulting α-(acyloxy)alkyl radical intermediates were rarely used to create another carbon–carbon bond, with the obvious exception of oligomerizations and polymerizations. We have found that by switching to the corresponding xanthates **13**, practically all the limitations discussed above could be lifted, resulting in an unusually powerful synthetic tool. The present short review will hopefully provide the reader with an idea of the scope of this chemistry.

**Scheme 2:**
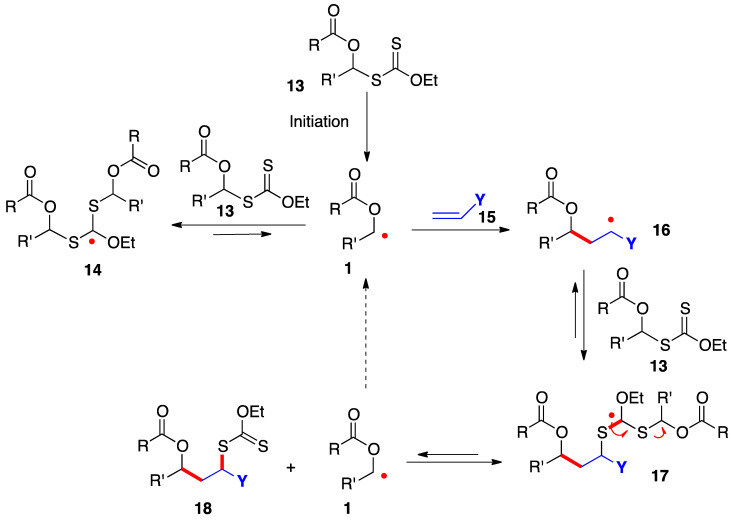
Mechanism of addition of α-(acyloxy)alkyl xanthates to alkenes.

The radical addition of xanthates **13** to alkenes **16** proceeds by the simplified mechanism outlined in Scheme 2 [7,8,9,10]. Thus, radical **1**, generated in an initiation step, is rapidly captured by the starting xanthate to give adduct radical **14**; however, this step is reversible and degenerate and does not consume radical **1**, which now acquires enough lifetime to allow it to add even to electronically unbiased alkene **15**. The resulting adduct **16** in turn is reversibly intercepted by starting xanthate **13** to provide addition product **18** by fragmentation of intermediate **17**. This sequence regenerates starting radical **1** to propagate the chain. The new carbon–carbon and carbon–sulfur bonds created in this process are colored in red. 

In addition to providing key radicals **1** and **16** with extended lifetime, highly stabilized and bulky species **14** and **17** act as reservoirs for these radicals and lower considerably their absolute concentration in the medium. The consequences are less complications from unwanted radical-radical interactions and a greater scope as regards poorly reactive alkene traps. The actual mechanistic picture is in fact much more sophisticated than is conveyed in Scheme 2 and the interested reader is directed to references [9,10] for a more extensive discussion. It is also worth mentioning that while the sequence in Scheme 2 shows the addition to an alkene, the extended lifetime of the intermediate radicals can often be exploited to overcome the slow kinetics of other radical transformations, such as fragmentations, unusual ring-closures, cyclizations and additions onto aromatic derivatives, and inter- and intra-molecular hydrogen atom abstractions.

From a practical standpoint, this manner of generating and capturing radicals offers many advantages. It uses cheap, readily available, and non-toxic starting materials and reagents; it is metal-free, and especially tin-free, even though certain metal complexes can be used as photoredox initiators; it tolerates numerous functional groups and solvents, including water, and can be performed at high concentrations and even without solvent; last, but not least, almost all types of carbon centered radicals, as well as nitrogen, oxygen, sulfur, phosphorus, and even stannyl radicals can be generated by this chemistry. 

## 2. Synthesis of *S*-α-(Acyloxy)alkyl Xanthates

**Scheme 3:**
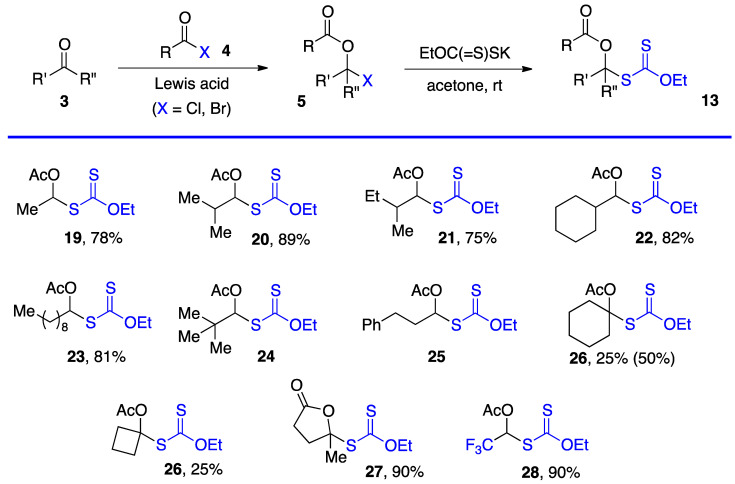
Synthesis of α-(acyloxy)alkyl xanthates from aldehydes and ketones [4,11,12,13,14].

We have employed two methods to obtain *S*-α-(acyloxy)alkyl xanthates **13**, but various other approaches can be envisaged. The most direct is by substitution of α-(acyloxy)alkyl halides **5** by a xanthate salt. The chlorides (**5**, X = Cl) are most common and are prepared from aldehydes and, but to a lesser extent, ketones by reaction with an acid chloride under catalysis by a Lewis acid, most commonly zinc chloride [1,2]. Xanthates made by this route are assembled in Scheme 3 [4,11,12,13,14] (note that the same numbers **3**, **5**, and **13** for the generic structures in Scheme 1, Scheme 2 and Scheme 3 are used are for both aldehydes and ketone derived compounds). Examples **24** and **25** are taken from a study by Lee and Kim (but no yields are given) [4]. Xanthates **27** and **28** were prepared by somewhat different routes. The former is derived from levulinic acid by treatment with neat thionyl chloride followed by reaction with potassium *O*-ethyl xanthate [11]. For the latter, the steps were in a way reversed. First commercial methyl hemiacetal of trifluoroacetaldehyde is treated with the xanthate salt and cold sulfuric acid and the resulting geminal xanthyl alcohol acetylated with acetic anhydride with catalysis by sulfuric acid [13,14].

**Scheme 4:**
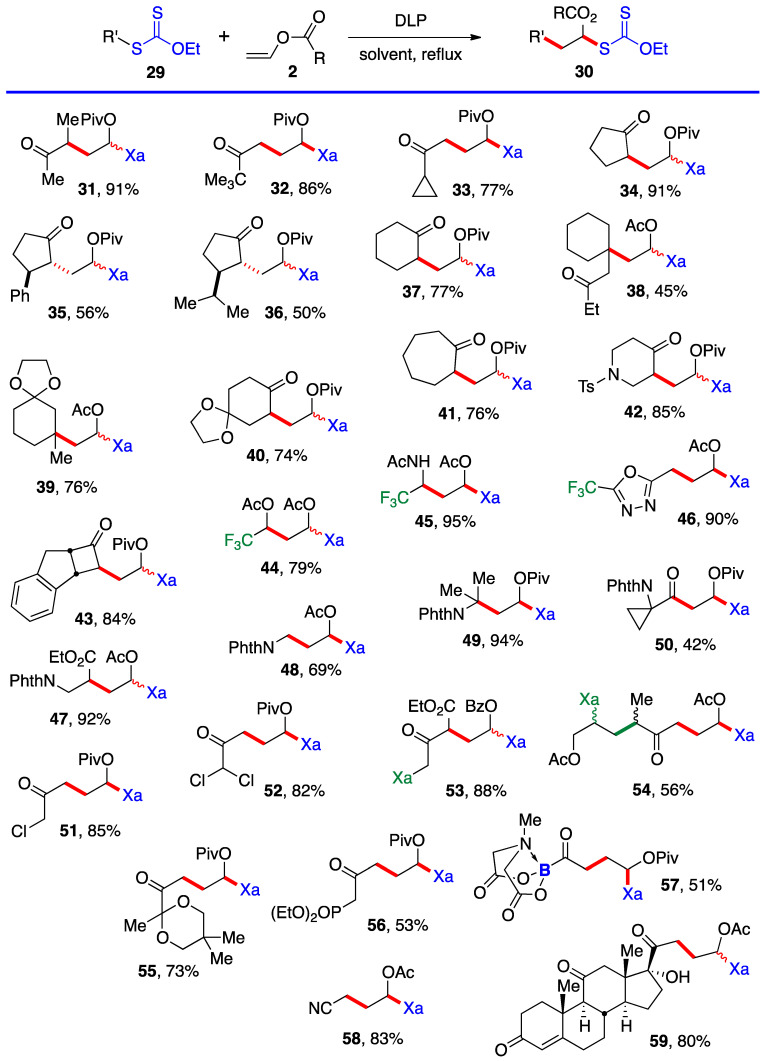
Synthesis of aliphatic and alicyclic α-(acyloxy)alkyl xanthates by radical addition to vinyl esters. Xa = -SC(=S)OEt; Piv = pivalate; Bz = benzoate; PhthN = phthalimido [13,15,16,17,18,19,20,21,22,23,24,25,26,27,28,29,30,31].

This first method is limited to aldehydes and to ketones that can withstand the strongly acidic conditions needed to form the α-(acyloxy)alkyl halides **5**. Xanthates **19**–**28** in Scheme 3 are thus relatively simple unfunctionalized derivatives. Examples **24** and **25** are from the article by Lee and Kim [4] who, unfortunately, did not record the yield. The second approach is by the radical addition of a xanthate to a vinyl ester, as shown in Scheme 4. This is an infinitely more powerful strategy because of the large number of available xanthates and the mild experimental conditions that accommodate many functional groups. Examples **31**–**59** assembled in Scheme 4 concern aliphatic and alicyclic derivatives (the literature reference where each compound is described is given in blue after the yield). The additions are simply accomplished by heating the xanthate and the vinyl ester in the solvent [most often ethyl acetate EtOAc, 1,2-dichloroethane (1,2-DCE), or cyclohexane] under an inert atmosphere at typically a 1 M concentration and adding the DLP portion-wise to initiate the process (DLP is di-lauroyl peroxide, also sold under lauroyl peroxide, Laurox^®^ or Luperox^®^). Upon heating to approximately 80 °C, DLP decomposes with a half-life of 1–2 h to give, after extrusion of CO_2_, primary undecyl radicals. These reactive radicals rapidly add to the thiocarbonyl group of the starting xanthate **13** to generate the more stable radical **1** by an addition–fragmentation analogous to the regeneration of radical **1** by reaction of adduct radical **16** with xanthate **13**. In Scheme 4 and in following schemes, wherever applicable, the diastereomeric ratio is approximately 1:1 unless otherwise indicated.

Ketones, protected or not, esters, nitriles, latent amines masked as phthalimides, phosphonates, nitriles, boronates, etc., can be present. α-Chloro- and α-dichloro-ketones **51** and **52** are particularly noteworthy in view of the sensitivity of these reactive motifs, especially the latter. In the case of di-xanthate **53**, the xanthate next to the ketone (in green) can be used to accomplish a regioselective second radical addition to various alkenes, if so desired, without complications from the other xanthate (in blue). The radical derived from the “green” xanthate is stabilized by conjugation with the ketone and is easier to generate than the radical from the “blue” xanthate, which is the precursor of a less stabilized radical. This difference in relative stabilities is a powerful handle to control the order of additions. Indeed, this is how compound **54** was obtained, first by addition to allyl acetate (bonds colored in green) followed by the addition to vinyl acetate (bonds in red). Xanthates are unique in allowing a modular construction of densely functionalized complex structures by implementing multiple carbon–carbon bond forming processes.

**Scheme 5:**
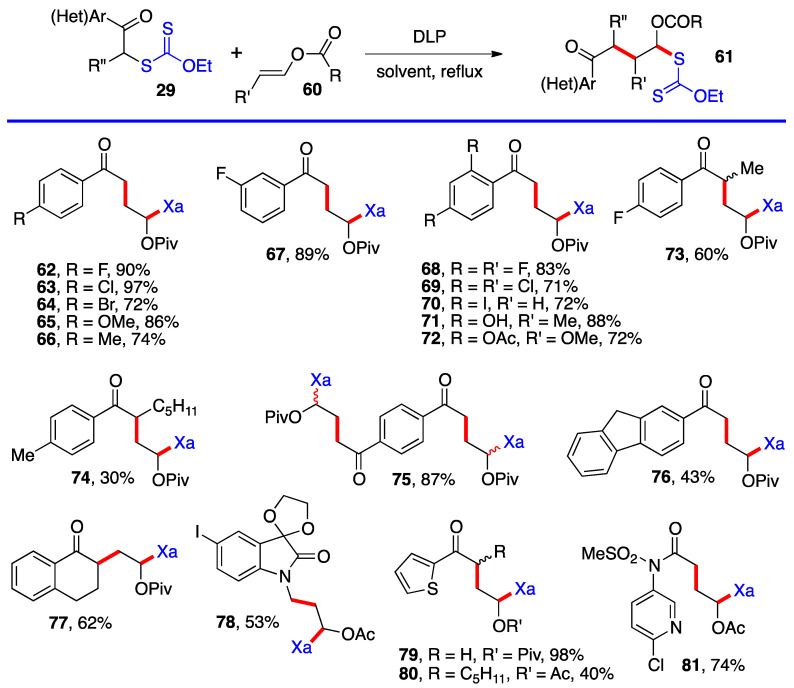
Synthesis of (hetero)aromatic α-(acyloxy)alkyl xanthates by radical addition to vinyl esters [15,16,32,33,34,35,36,37].

Aromatic and heteroaromatic derivatives **62**–**81** of generic structure **61**, with the exception of compound **78** which is not derived from a ketonyl xanthate, are presented in Scheme 5. As will be shown later in this review, many of these adducts were used to prepare tetralones and naphthalenes by using the xanthate group to accomplish a ring-closure onto the aromatic or heteroaromatic ring.

**Scheme 6:**
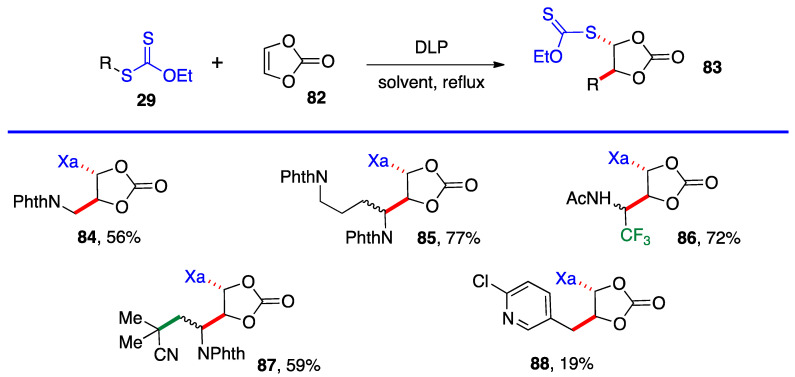
Synthesis of α-(acyloxy)alkyl xanthates by radical addition to vinylidene carbonate [20,23,38,39,40].

Most of the additions in Scheme 4 and Scheme 5 were performed on vinyl pivalate, in part because the yields are generally somewhat higher than with vinyl acetate, presumably because unwanted oligomerization is slower and competes less with the desired mono-addition. Substituted alkenyl esters (**60**, R’≠ H) react sluggishly and the yields are significantly lower, as illustrated by adducts **74** and **80**. A special case is that of vinylidene carbonate **82** (Scheme 6). It has a reasonable reactivity and leads to interesting, highly functional *trans* adducts **83** which can be converted into protected vicinal diols. Five examples are displayed in Scheme 5. In the case of adduct **87**, the xanthate used to react with vinylidene carbonate **82** is itself derived by addition of *O*-ethyl-*S*-(1-cyano-1-methyl)propyl xanthate to vinyl phthalimide. The bond formed in this addition is colored in green.

## 3. Radical Additions of *S*-α-(Acyloxy)alkyl xanthates

**Scheme 7:**
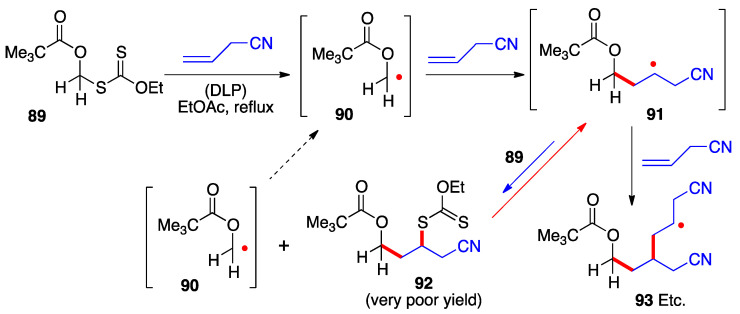
Failed addition of a primary α-(acyloxy)alkyl xanthate due to unfavorable relative stabilities of the intermediate radicals.

The examples in the preceding schemes give an idea of the range of functionalized structures of xanthates that can act as precursors for the corresponding α-(acyloxy)alkyl radicals. Their capture by an external alkene proceeds by the radical chain mechanism presented in Scheme 2. Implicit in this mechanistic manifold is the need for the starting radical **1** to be more stable than adduct radical **16** (neglecting for the moment polar factors), for otherwise the equilibrium between these two radicals, passing through intermediate **17**, will favor the latter. The consequence is a significant unwanted oligomerization by further addition of adduct radical **16** to alkene **15**. This untoward situation obtains in the case of the α-pivaloxymethyl xanthate **89** (Scheme 7) [23]. Its addition to allyl cyanide hardly produces any of the corresponding adduct **92** because primary radical **90** is not sufficiently stabilized by the pivaloxy group alone to make it more stable than adduct radical **91**. The unfavorable equilibrium causes adduct radical **91** to accumulate leading to the predominant formation of oligomers (radical **93**) and other side-products.

**Scheme 8:**
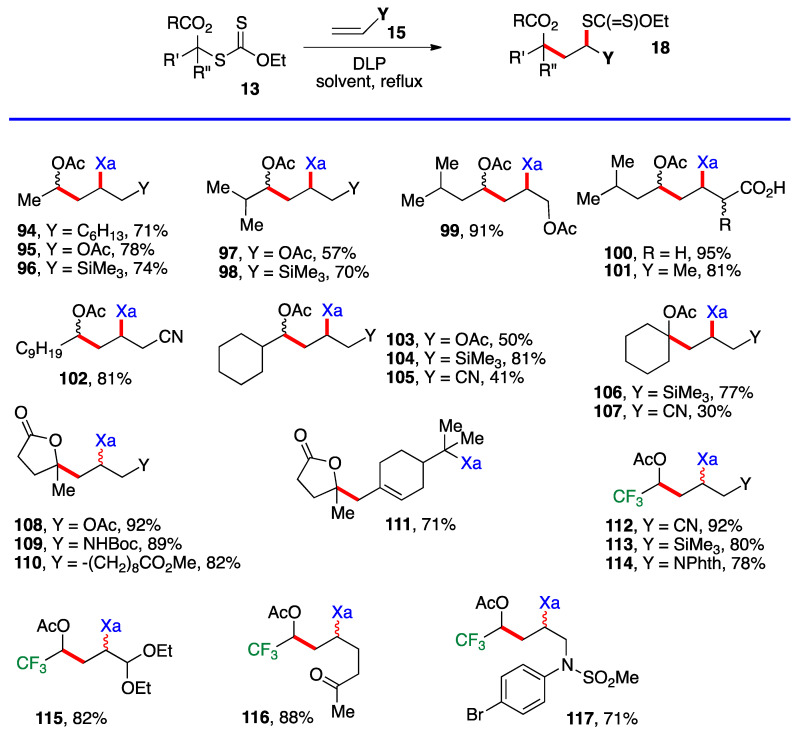
Addition of α-(acyloxy)alkyl xanthates to various alkenes [11,12,13].

However, the difference in stability is slight and, once a substituent is introduced (i. e. **1**, R’ ≠ H), the relative stabilities are reversed. The desired chain is now correctly propagated and furnishes the requisite addition products **18**, as summarized by the generic equation at the top of Scheme 8. The reactions are carried out in the same manner as for the additions in Scheme 4, Scheme 5 and Scheme 6, namely by merely heating the xanthate and the alkene with portion-wise addition of the peroxide initiator. Examples **94**–**117** in Scheme 8 derive from the simpler xanthates in Scheme 3 (with the exception of cyclobutyl xanthate **26**), which were obtained by chloroacetylation of aldehydes and ketones. Many of the synthetically most useful functional groups are tolerated on the alkene partner. The protected amines in examples **109** and **114**, and the protected aldehyde and the free ketone in compounds **115** and **116** are especially noteworthy. Derivative **111** results from the addition–fragmentation of xanthate **29** to β-pinene.

**Scheme 9:**
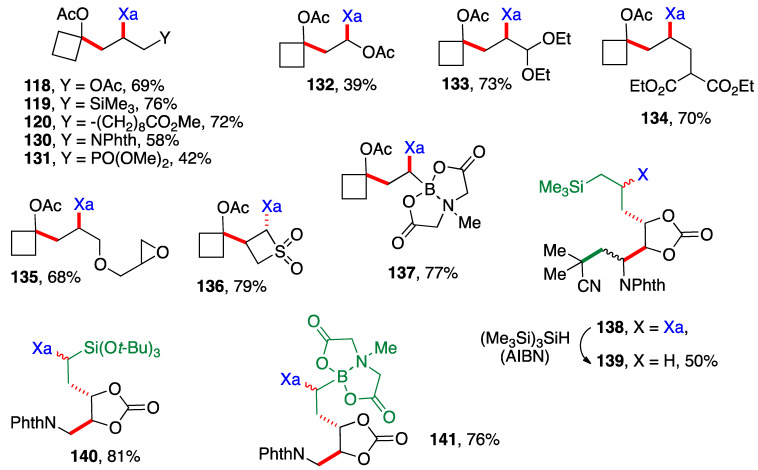
Further examples of addition of α-(acyloxy)alkyl xanthates to various alkenes [12,39,41,42].

Scheme 9 presents examples of additions of cyclobutyl xanthate **26** (Scheme 3) and xanthates bearing the cyclic carbonate motif found in Scheme 6. The alkene partners are similar to those in Scheme 8, with a few additional interesting ones, namely the presence of an epoxide in adduct **135**, a 4-membered sulfone in adduct **136** and a (MIDA)boronate in adducts **137** and **141** (MIDA = *N*-methyliminodiacetyl). The cyclic carbonate derivative **138** is in fact the result of three intermolecular additions with the creation of three new C—C bonds and one C—S bond (colored in green and red) and the modular combination of four different molecules. The xanthate was reductively removed to simplify the structure and spectral assignments. The yield given for sulfur-free product **139** is for the two steps which were telescoped. Incidentally, note that adduct **132** arises from addition of xanthate **26** to vinyl acetate and is thus also a precursor to an α-(acyloxy)alkyl radical. It could therefore in principle participate in a second radical addition if so desired. The moderate yield of adduct **132** is due to competing oligomerization.

## 4. Further Additions and Applications of *S*-α-(acyloxy)alkyl Xanthates

**Scheme 10:**
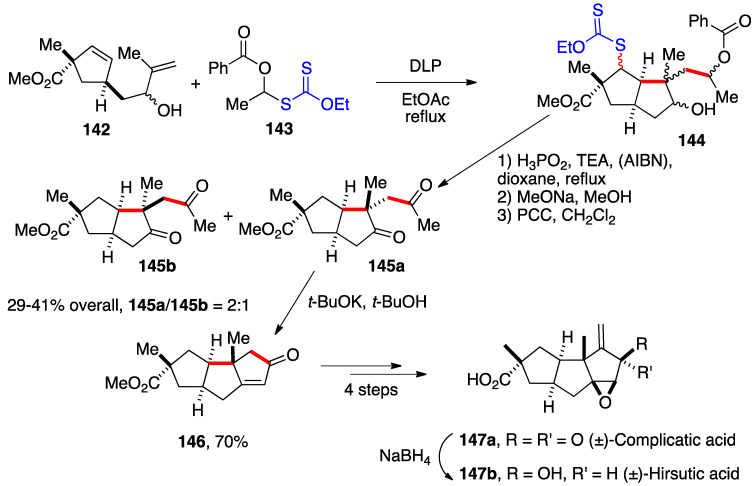
A formal total synthesis of (±)-complicatic and hirsutic acids [43,44].

Access to convenient precursors to α-(acyloxy)alkyl radicals and the ability to accomplish intermolecular additions to both activated and non-activated alkenes opens numerous synthetic opportunities. The sequence in Scheme 10 was used in a formal synthesis of (±)-complicatic and hirsutic acids [43]. The acetaldehyde derived xanthate **143** reacts with diene **142** to give bicycle **144** by consecutive addition, cyclization, and xanthate transfer. Reductive removal of the xanthate using Barton’s hypophosphorous method, saponification, and oxidation with pyridinium chlorochromate (PCC) removes most of the chiral centers and results in the formation of two epimers **145a** and **145b** in a 2:1 ratio in favor of the desired epimer. The use of the bulkier benzoate **143** instead of acetate analogue **19** is to alter the final epimeric ratio in the right direction. The intermediates in this sequence were not purified as they consisted of complex diastereomeric mixtures. Treatment of the major epimer with base induces a Robinson-type annulation to give triquinane **146**. This compound had been converted by Matsumoto and co-workers into both (±)-complicatic and hirsutic acids **147a** and **147b** in 4 and 5 steps, respectively [44].

**Scheme 11:**
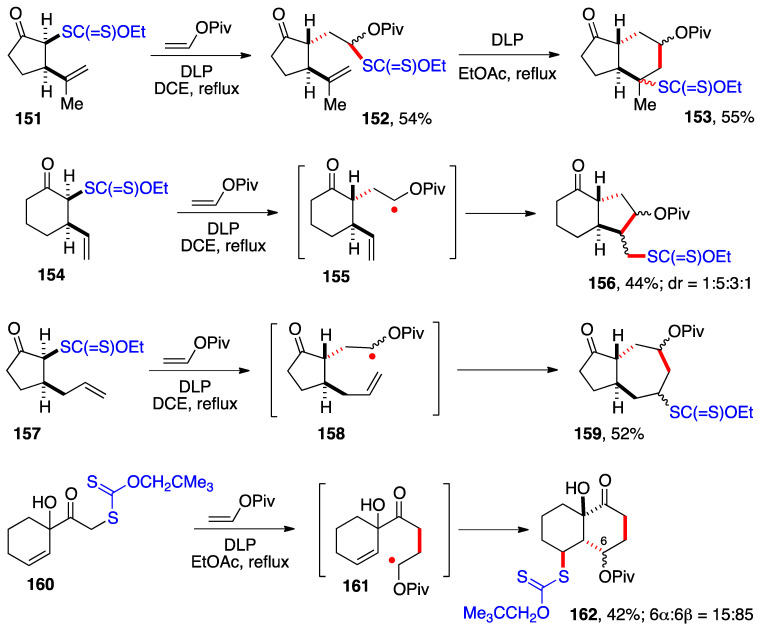
Bicyclic structures by generation and ring-closure of α-(acyloxy)alkyl radicals.

The generation of α-(acyloxy)alkyl radicals by reaction of xanthates with vinyl esters and their capture by an internal alkene represents a versatile route to polycyclic architecture. The transformations displayed in Scheme 11 showcase the formation of 5-, 6-, and 7-membered rings and the construction of various bicyclic structures related to terpenes. In the first, adduct **152** from the addition of xanthate **151** to vinyl pivalate was isolated and the 6-*endo* cyclization leading to *trans*-hydrindanone **153** accomplished in a separate step [17]. In the second, *trans*-hydrindanone **156** was prepared from xanthate **154** by a 5-*exo* cyclization of intermediate radical **155**, without isolation of the intermediate adduct [17]. *Trans*-hydrindanes are subunits of most natural steroids. They are thermodynamically less stable than the corresponding *cis*-epimers and their synthesis is rarely trivial. The two routes underscore the flexibility and modularity provided by this chemistry of xanthates.

The third and fourth examples concern the formation of *trans*-perhydroazulenone **159** and *cis*-decalin **162** from xanthates **157** and **160** by 7-*endo* and 6-*exo* cyclization of intermediate α-(acyloxy)alkyl radicals **158** and **161**, respectively [17,45]. Many more structural variations can be envisaged and, moreover, the pivalate group can be considered a latent ketone, and this opens up numerous possibilities for introducing further complexity. The presence of the xanthate is a particularly useful handle for additional modifications, even though in most cases it was simply reductively removed.

**Scheme 12:**
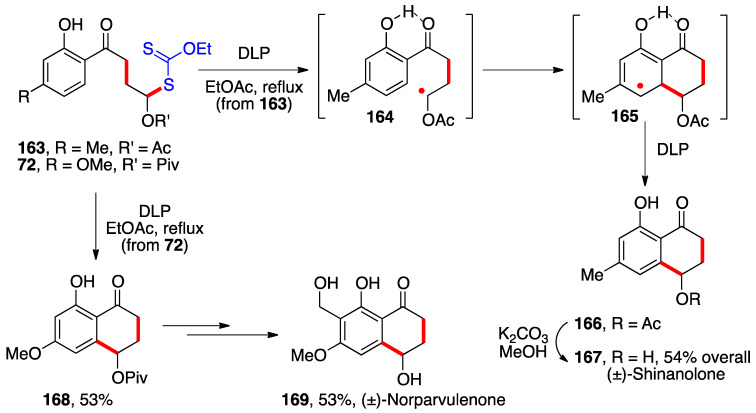
Total synthesis of 4-hydroxytetralone natural products.

The α-(acyloxy)alkyl radicals generated from the aromatic adducts pictured in Scheme 5 can undergo ring closures on the aromatic motif. Two applications of this variant are outlined in Scheme 12. In the first, adduct **163** to vinyl acetate can be converted into tetralone **166** by simply heating with stoichiometric amounts of peroxide [33]. Under these conditions, α-(acetoxy)alkyl radical **164** is recreated and, in the absence of a competing external trap (apart from the solvent), undergoes cyclization into cyclohexadienyl radical **165**. This step is reversible and entails temporary loss of aromaticity, which is restored by oxidation of radical **165** to the corresponding cation (not shown) by electron transfer to the peroxide and subsequent loss of a proton. In this particular case, the hydrogen bonding between the ketone and the ortho hydroxy group freezes the conformation in a geometry that is propitious for ring closure. Saponification of the pendant acetate furnishes (±)-shinanolone **167**.

A similar cyclization of xanthate **72** affords tetralone **168**, which was converted into (±)-norparvulenone in three steps [33,34]. These two total syntheses are not only the shortest to these natural products, but they prompt two observations. The first is the possibility of accomplishing both the intermolecular addition to vinyl pivalate and the cyclization in the presence of the free phenol. Phenols are well-known radical inhibitors [46], yet the xanthate chemistry proceeds normally. A partial explanation is that the hydrogen bonding slows down in fact the rate of the phenolic hydrogen abstraction [47], allowing the desired radical steps to take place. The second observation is the fact that the cyclization onto the aromatic ring can be viewed as the equivalent of an intramolecular Friedel–Crafts reaction, but accomplished under much milder, neutral conditions. Indeed, the substrates in Scheme 12 would not survive typical Friedel–Crafts conditions. This radical cyclization onto aromatics and heteroaromatics deserves a greater attention from synthetic chemists.

**Scheme 13:**
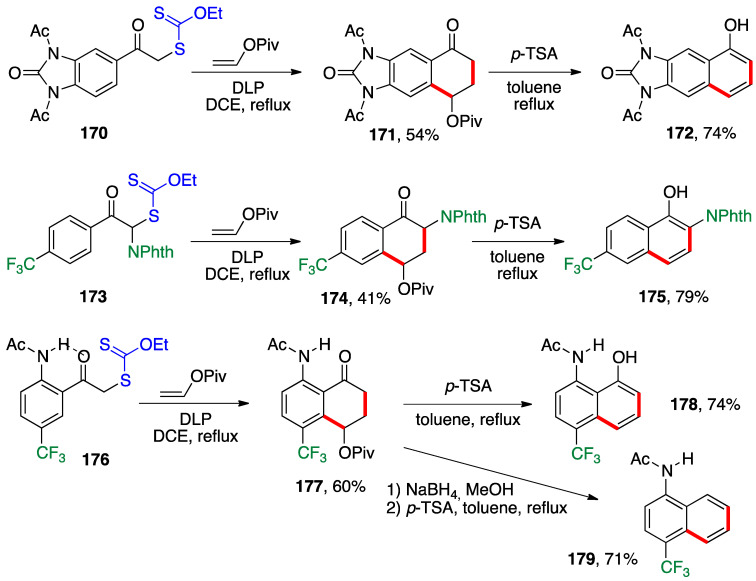
A convergent route to naphthalenes.

The ability to access 4-acyloxytetralones by radical addition of phenacyl xanthates to vinyl esters followed by ring closure onto the aromatic ring opens up another synthetic application, namely the synthesis of naphthalenes by acid-catalyzed elimination of the acyloxy group. This approach is illustrated by the examples in Scheme 13 [48,49,50]. The addition and cyclization are performed in the same flask since both steps use DLP as both the initiator and stoichiometric oxidant. In this manner, the reaction of xanthate **170** with vinyl pivalate provides tetralone **171** and heating with acid induces aromatization into naphthol **172** [48]. Amino-protected 1,2-naphtholamine **175** was prepared similarly from xanthate **173** via tetralone **174** [48]. The addition–cyclization starting with xanthate **176** benefits from an intramolecular hydrogen bonding in the same manner as for the phenolic derivatives in Scheme 12 [49]. This allows the synthesis of *N*-acetyl aminonaphthol **178**. Reduction of the ketone in intermediate **177** prior to treatment with acid furnishes *N*-acetylamino-naphthalene **179** [49]. Many reactions in addition to reduction can in fact be performed on the intermediate tetralones before the elimination of the relatively robust pivalate group. These include bromination, addition of various organometallics to the ketone, and Wittig-type condensations. This results in a considerable expansion of the scope, making this method one of the most versatile routes to naphthalenes. Many additional examples can be found in a recent review on the subject [50].

**Scheme 14:**
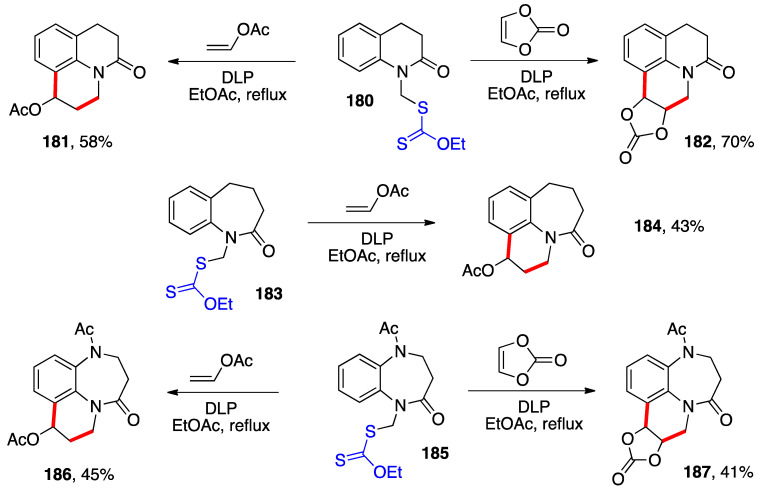
Further additions to vinyl esters and cyclizations onto aromatics.

The same strategy can be used to build other polycyclic aromatic structures. Examples of various tetrahydroquinolines are deployed in Scheme 14 [36]. In these cases, too, the intermediate adducts were not isolated and the addition–cyclization was accomplished simultaneously. The presence of the acetate and carbonate groups in the products opens numerous possibilities for further transformation and diversification. This is an important asset for compounds that could be of pharmaceutical interests (note the structural resemblance to benzazepinones of derivatives **184**, **186**, and **187**).

**Scheme 15:**
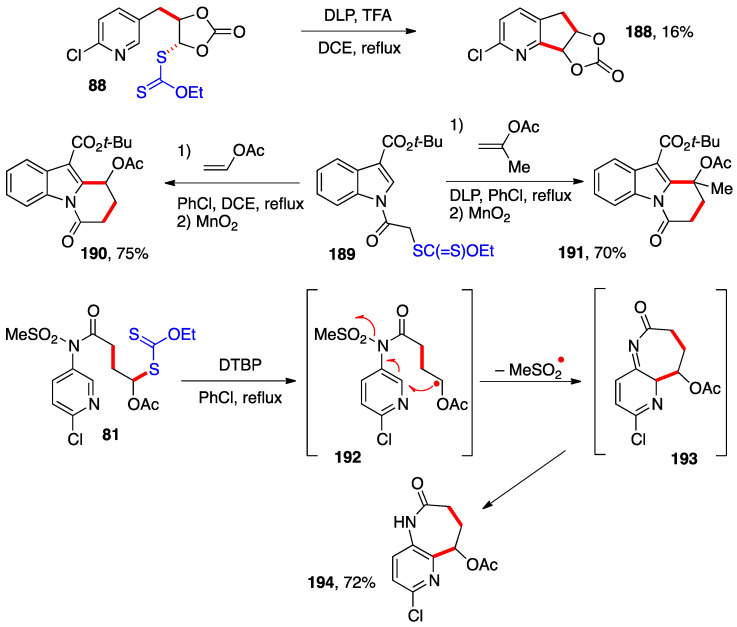
Additions to vinyl esters and cyclizations onto heteroaromatics.

Radical addition–cyclization can be accomplished on heteroaromatic derivatives (Scheme 15). Xanthate **88** is made by addition of *S*-2-chloro(pyridyl-5-methyl) xanthate to vinylidene carbonate (Scheme 6). Further treatment with DLP in the presence of trifluoroacetic acid cause ring closure into azaindane **188**, albeit in poor yield [40]. The weak performance is almost certainly due to the strain in the product caused by the fusion of two relatively flat 5-membered rings. Similar cyclizations with adducts of open-chain alkenes proceed significantly more efficiently. The protonation of the pyridine ring by the trifluoroacetic acid enhances the cyclization, in line with the early observations of Minisci.

Starting with xanthate **189**, addition–cyclization with vinyl acetate and isopropenyl acetate furnish tricyclic indole derivatives **190** and **191**, respectively, where a new 6-membered ring has been created [51]. The presence of a substituent on C–3 of the indole ring is necessary for an efficient cyclization. The electron-withdrawing ester group in the present case, however, slows the rearomatization step through oxidation with the peroxide. It is therefore necessary to complete the aromatization by addition of manganese dioxide to the reaction mixture.

The cyclization of adduct **81** to produce azepinone **194** is particularly interesting [37]. It is performed at the higher temperature of refluxing chlorobenzene (130 °C) in the presence of di-*t*-butyl peroxide (DTBP) and leads to the formation of a 7-membered ring. The ring closure of intermediate radical **192** proceeds with concomitant extrusion of a methylsulfonyl radical to give imine **193**, which then tautomerizes to the desired product. This elimination restores the aromaticity of the pyridine ring and at the same time deprotects the nitrogen to give azepinone **194** with an unsubstituted nitrogen. The presence of a free site on the secondary lactam should allow the introduction of numerous groups and the building of libraries for biological testing. All the products in Scheme 15 are medicinally relevant.

**Scheme 16:**
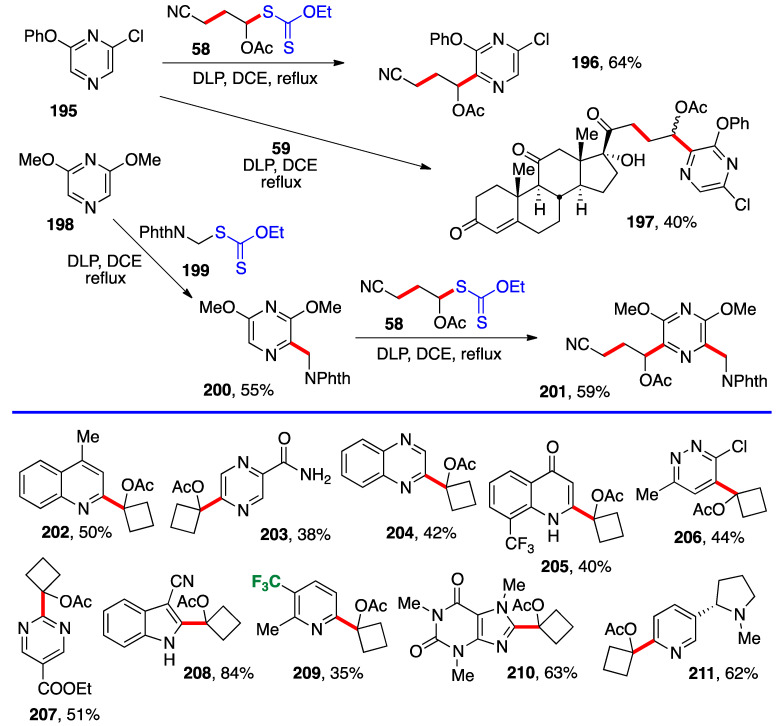
Intermolecular additions of α-(acyloxy)alkyl radicals onto heteroaromatics.

Intermolecular addition to certain heteroaromatics is often possible with xanthates, and the addition of α-(acyloxy)alkyl radicals is no exception. This convergent Minisci-type approach can be quite powerful for the late-stage modification of biologically active substances and for the optimization of their pharmacological profile. This is illustrated by the transformations involving pyrazine substrates displayed in Scheme 16. Thus, the reaction of xanthates **58** and **59** with pyrazine **195** proceeds regioselectively to give compounds **196** and **197 [31]**. The latter example is quite spectacular, as it would be exceedingly tedious to make by other chemistries. In the case of symmetrical pyrazine **198**, successive reactions with xanthates **199** and **58** furnish unsymmetrical pyrazine **201 [31]**.

Examples of additions of cyclobutyl xanthate **26** (Scheme 3) to a variety of heteraromatics are also assembled in Scheme 16 [12]. The yields are variable, and basic heteroaromatics required activation by addition of camphorsulfonic acid; nevertheless, a collection of otherwise difficultly accessible series of compounds could be rapidly prepared for an eventual biological screening. Interestingly, adducts **210** and **211** derive from caffeine and nicotine, respectively.

**Scheme 17:**
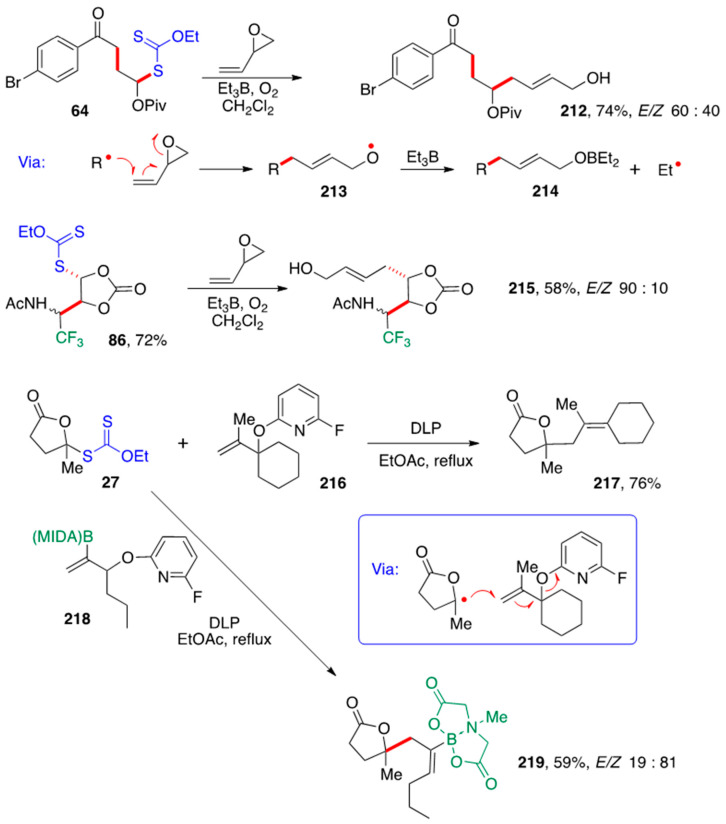
Addition–fragmentation involving α-(acyloxy)alkyl radicals [18].

α-(Acyloxy)alkyl radicals can participate in different reaction modes, namely a variety of addition–fragmentation (Please keep “addition-fragmentation”) processes. The first transformation in Scheme 17 is an example involving a vinyl epoxide [52]. Thus, portion-wise addition of triethylborane to a stirring solution of xanthate **64** and the monoepoxide of butadiene in dichloromethane at room temperature and injection of small quantities of air results in the formation allylic alcohol **212** in good yield. It is interesting that, in the present case, one obtains a regioselectively protected diol: an unprotected allylic alcohol and a pivalate protected homoallylic alcohol.

This reaction is based on earlier studies by Brown [53] and Oshima [54] and proceeds by way of alkoxy radical **213**, which is captured by the triethylborane to give borinate **214** and an ethyl radical that propagates the chain by attacking the starting xanthate. The borinate is hydrolyzed during workup. The same reaction starting from xanthate **86** furnishes allylic alcohol **215**. The allylation of xanthates with vinyl epoxides mediated by triethylborane has a much broader scope than earlier work with other substrates such as iodides [54].

We discovered an even more powerful allylation method using allylic alcohols as the radical allylating agents. Normally, β-fragmentation of C–O bonds by homolysis is difficult because such bonds are strong and the alkoxy radical generated is a high-energy species. This feature is very valuable for the modification of carbohydrates where C–O bonds are abundant. Epoxides constitute of course a notable exception, as the ring opening to give alkoxy radicals is exceedingly fast. β-Lactones also rapidly fragment [55]. In both cases, the driving force is the relief of the strain contained in such small rings. With unstrained acyloxy groups, the b-fragmentation is too slow to be synthetically useful. However, we found that with fluoropyridyloxy derivatives, the fragmentation is sufficiently accelerated that, in combination with the relatively long lifetime of radicals produced via xanthates, it becomes feasible, with far reaching consequences [56]. Two examples are shown in Scheme 17. In the first, xanthate **27** reacts with allyl fluoropyridyloxy derivative **216** by an addition–fragmentation sequence (see box in Scheme 17) to give tetrasubstituted alkene **217 [57]**. In the second, addition–fragmentation to vinyl (MIDA)boronate **218** furnishes compound **219** with a 4:1 stereoselectivity in favor of the less hindered *Z*-isomer [58].

**Scheme 18:**
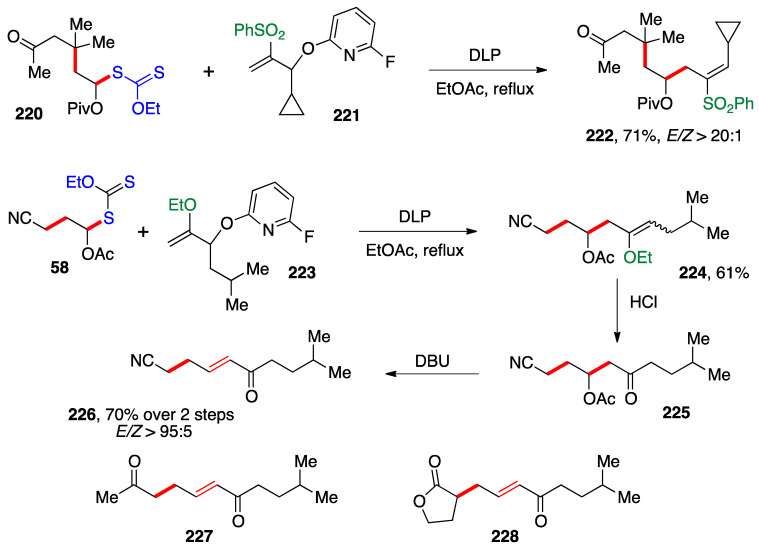
Further examples of addition–fragmentation of α-(acyloxy)alkyl radicals.

The formation of tetrasubstituted alkenes, such as **217**, is not easy to accomplish using classical reactions such as the Wittig condensation. The present approach provides a simple solution to this synthetic difficulty. It also allows the construction of functional alkenes which could then be further elaborated, through the powerful Suzuki coupling in the case of vinyl (MIDA)boronate **218**. Other interesting examples are provided in Scheme 18. For instance, xanthate **220**, itself made by addition of mesityl oxide derived xanthate to vinyl pivalate, reacts with fluoropyridyloxy derivative **221** leads to sulfone **222** with essentially total stereoselectivity [59]. Such vinylsulfones can be reductively desulfonylated with retention of configuration using sodium dithionite (the Julia method), thus opening a convenient access to *Z*-alkenes.

By placing an ethoxy group on the alkene partner, as in compound **223**, then the addition–fragmentation of an α-(acyloxy)alkyl xanthate such as **58** leads to ethoxyvinyl adduct **224**, which can be hydrolyzed with aqueous acid into ketone **225 [60]**. Treatment with DBU then induces elimination of the acetate to give α,β-unsaturated ketone **226**. Thus, both saturated and unsaturated ketones can be obtained by this approach. Two additional examples, **227** and **228**, are shown at the bottom of Scheme 18.

**Scheme 19:**
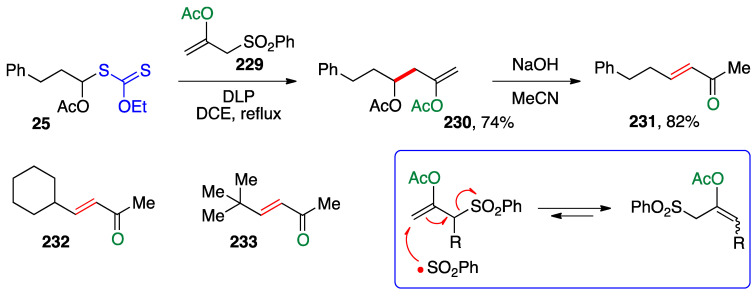
Addition–fragmentation of to allylsulfones α-(acyloxy)alkyl radicals.

A similar strategy was applied earlier by Lee and Kim using the more traditional allyl sulfone **229** as the allylating agent (Scheme 19) [4]. Its reaction with xanthate **25** leads to enol acetate **230** which, upon saponification gives directly enone **231**. Enones **232** and **233** were prepared by the same procedure. This approach is efficient and should work equally with more elaborate xanthate precursors of α-(acyloxy)alkyl radicals. It suffers, however, from limitations in the structural diversity of the allylating agent. Allylsulfones substituted at the carbon bearing the sulfonyl group are prone to isomerization by a radical chain process, as indicated in the box in Scheme 19 [61].

**Scheme 20:**
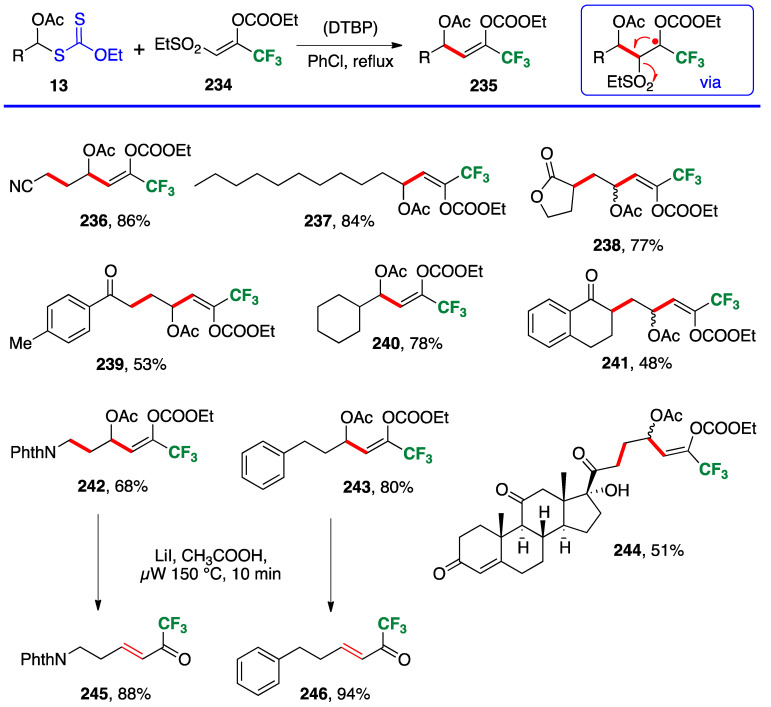
Addition–fragmentation of α-(acyloxy)alkyl radicals to vinylsulfones.

Vinylation using vinyl sulfones is also possible. This variant is illustrated by the synthesis of a variety of trifluoromethyl enol carbonates **236**–**244** using readily available reagent **234** [62]. The reaction is conducted in refluxing chlorobenzene with di-*t*-butyl peroxide as the initiator, and proceeds by an addition–fragmentation (see box at the top of Scheme 20). The ethylsulfonyl radical that is extruded in the fragmentation step loses sulfur dioxide to give an ethyl radical that propagates the chain. The adducts are latent enones that can be revealed by heating briefly in a microwave oven with lithium iodide in acetic acid. Two α,β-unsaturated trifluoromethylketones, **245** and **246**, were also thus obtained.

## 5. Concluding Remarks

**Scheme 21:**
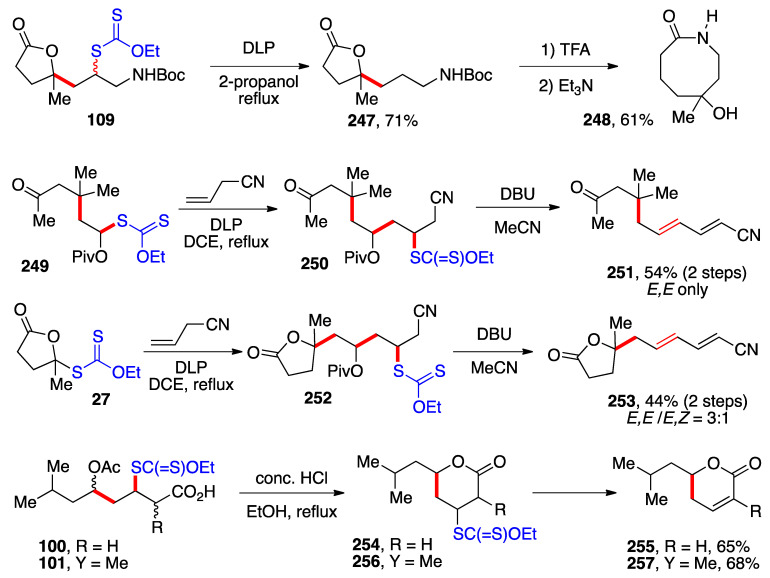
Examples of further transformations of adducts of α-(acyloxy)alkyl radicals.

The addition of xanthates to vinyl esters already represents a powerful synthetic tool [63]. For instance, adducts from α-ketonyl xanthates are convenient precursors for pyrroles and thiophenes [15,16]. The ability to use these adducts as precursors of α-(acyloxy)alkyl radicals in a second inter- or intra-molecular addition allows a rapid increase in complexity and opens further synthetic opportunities through subsequent ionic transformations. Some examples are assembled in Scheme 21. These also include cases where the starting xanthate was prepared directly from an aldehyde or a ketone. Thus, reductive removal of the xanthate from adduct **109** and unmasking of the amine causes spontaneous ring closure to give 8-membered lactam **248** [11]. Adducts **250** and **252** from the addition of xanthates **249** and **27** to allylcyanide undergo base induced b-elimination of both the xanthate and the pivalate groups upon exposure to DBU to afford dienenitriles **251** and **253**, respectively. Finally, heating adducts **100** and **101** with concentrated HCl in refluxing ethanol gives rise to unsaturated lactones **255** and **257** via lactones **254** and **256** from which the xanthate is eliminated in a slower step. It is worth pointing out the much greater stability of a xanthate to acidic condition as compared to an ester.

The foregoing examples give an idea of the synthetic possibilities of α-(acyloxy)alkyl radicals **1** generated from their xanthate precursors **13**. This path to ester protected carbinols is complementary to ionic routes, which rely heavily on the addition of organometallic nucleophiles to aldehydes and ketones. In view of the mild nucleophilic character of α-(acyloxy)alkyl radicals **1**, the present approach represents in a sense an “umpolung” of aldehydes and ketones since these are normally the electrophilic or acceptor partner in ionic additions. However, α-(acyloxy)alkyl radicals **1** exhibit a broader reactivity because they are able to add to electrophilic partners with complementary polarity and to unactivated, electronically unbiased alkenes. This is made possible by the increased lifetime provided by the xanthate to the various active radicals and the lowering of their absolute concentration in the medium, which limits unwanted radical-radical interactions and indirectly favors the desired addition to the alkene despite the relatively slow rates. Another enormous advantage is the tolerance for polar functional group which usually require protection in ionic or organometallic settings. This is nicely encapsulated by the success of reactions leading to compounds **197** and **244** (Scheme 16 and Scheme 20) and involving heavily functionalized steroid **59** (Scheme 4), itself prepared by addition of the xanthate precursor to vinyl acetate. Such compounds would be exceedingly hard to make by other chemistries.
